# Supplementary material for: A Likelihood Approach for Real-Time Calibration of Stochastic Compartmental Epidemic Models
Source: PLoS Comput Biol. 2017 Jan 17;13(1):e1005257. doi: 10.1371/journal.pcbi.1005257 (PMC5240920; doi:10.1371/journal.pcbi.1005257)
Supplement: S1 File — (TAR.GZ) [file pcbi.1005257.s014.tar.gz › HSPH_Online-SI-Revision/EnKF07/n100-mild/n100-mild_PF_EnKF-summery.pdf]

peak -8	R0	$1/\theta_2$	inf	inflog	newly	newlylog	Reff	AttackRate
mean	88.	147.7	33 811.3	1.8	14 520.	1.4	60.4	248.
median	89.1	103.4	20 648.4	2.1	7837.4	1.7	64.1	253.3
Quantile .1	69.2	51.1	542.9	0.6	247.9	0.3	32.4	95.1
Quantile .9	102.4	288.5	78 636.9	2.7	38 043.3	2.3	73.1	365.1
33	40	9						
length	50							

peak -4	R0	$1/\theta_2$	inf	inflog	newly	newlylog	Reff	AttackRate
mean	97.9	128.6	2785.	0.7	491.3	0.6	63.6	312.6
median	98.1	100.4	285.4	0.5	189.4	0.5	64.9	306.3
Quantile .1	84.1	39.7	83.4	0.2	101.6	0.3	52.5	236.4
Quantile .9	109.6	233.6	5708.3	1.6	1169.6	0.9	72.	381.2
20	35	0						
length	50							

peak	R0	$1/\theta_2$	inf	inflog	newly	newlylog	Reff	AttackRate
mean	99.4	150.9	120.1	0.5	107.	0.8	56.8	326.1
median	100.4	137.	108.8	0.5	100.2	0.8	55.4	311.8
Quantile .1	81.4	55.8	74.7	0.3	89.1	0.4	48.8	268.9
Quantile .9	114.4	266.8	168.1	0.8	131.1	1.1	66.3	401.
22	34	0						
length	50							

peak +4	R0	$1/\theta_2$	inf	inflog	newly	newlylog	Reff	AttackRate
mean	96.3	103.9	1356.6	0.7	860.4	0.6	52.6	329.2
median	94.8	95.1	654.6	0.7	561.1	0.6	51.3	327.6
Quantile .1	82.8	32.5	91.6	0.3	136.1	0.3	41.8	257.6
Quantile .9	112.4	182.8	1778.	1.	1126.2	0.8	61.6	417.7
23	19	0						
length	50							

peak +8	R0	$1/\theta_2$	inf	inflog	newly	newlylog	Reff	AttackRate
mean	101.3	173.5	106 266.	1.5	166 252.	1.3	58.3	315.2
median	100.6	147.2	4286.3	1.4	2674.8	1.1	59.7	299.5
Quantile .1	83.9	56.9	697.1	0.7	769.9	0.6	40.8	265.1
Quantile .9	114.2	325.1	40 003.7	2.4	15 720.5	1.9	72.	385.6
13	28	0						
length	50							
